# PP2A targeting: balancing anticancer potential and neurological risk

**DOI:** 10.3389/fcell.2026.1945801

**Published:** 2026-10-13

**Authors:** Margarita Chigriai, Nina Kraskovskaya, Anastasia Bolshakova, Natalia O. Porozova, Yuri B. Porozov, Milica Pešić

**Affiliations:** 1 Advitam Laboratory, Belgrade, Serbia; 2 Institute of Cytology RAS, St. Petersburg, Russia; 3 The Laboratory of Bio- and Chemoinformatics, School of Computer Science, Physics and Technology, HSE University, St. Petersburg, Russia; 4 Department of Neurobiology, Institute for Biological Research “Siniša Stanković” – National Institute of the Republic of Serbia, University of Belgrade, Belgrade, Serbia

**Keywords:** Alzheimer’s disease, cancer, LB100, neurodegeneration, neurodevelopment, oncogenic signaling, PP2A

## Abstract

Protein phosphatase 2A (PP2A) is a multifunctional serine/threonine phosphatase that regulates cell-cycle progression, oncogenic signaling, neuronal development, synaptic homeostasis, and protein proteostasis. Its dysregulation contributes to cancer, whereas impaired PP2A activity in the nervous system is linked to neurodevelopmental disorders and neurodegenerative proteinopathies, including tau and α-synuclein pathology. These divergent roles create both opportunities and safety challenges for PP2A-directed therapies. This review examines PP2A as a modular holoenzyme system whose subunit composition determines substrate specificity and context-dependent biological effects. We discuss PP2A dysfunction in cancer, including genetic and regulatory alterations affecting PP2A subunits, assembly, and endogenous inhibitors, and compare therapeutic PP2A activation with transient inhibition. The latter approach is exemplified by LB100, which sensitizes treatment-stressed tumor cells to chemotherapy, radiotherapy, or immunotherapy by disrupting checkpoint adaptation and stress recovery. We further evaluate the neurological implications of systemic phosphatase inhibition, LB100 conversion to the active metabolite endothall, clinical safety and pharmacokinetic data, and the limited brain-tumor penetration of intact LB100. We propose that the clinical utility of PP2A inhibition depends on defining a therapeutic corridor that integrates dosing schedule, parent-drug and metabolite exposure, tumor target engagement, biomarker-informed patient selection, and preservation of PP2A-dependent homeostasis in normal tissues, particularly the nervous system.

## 1 Introduction

According to the World Health Organisation, cancer is a leading death cause worldwide. Despite the enormous efforts made to fight cancer, the incidence statistics are only growing. One of the major challenges in cancer treatment is overcoming drug resistance, which can develop even in patients with a good original response to therapy (Groenendijk and Bernards, 2014; Pérez-Herrero and Fernández-Medarde, 2015; Aldea et al., 2021). Advanced tumors are highly heterogeneous and adaptable, so drug pressure quickly selects variants whose signaling has been rewired to bypass the treatment. Frequently the resistant cells restore the very pathway the drug blocks, through secondary mutations that reactivate it despite the drug’s presence (Dias et al., 2024a).

Recent studies have proposed an unconventional approach known as “paradoxical overactivation” (Dias et al., 2024a), which consists of the hyperactivation of oncogenic signaling, which leads to cellular homeostasis disruption, stress response pathways overload, and finally cell death promotion.

Protein phosphatase 2A (PP2A) is a serine/threonine phosphatase that is crucial in the mitosis progression and the cellular response to DNA damage.

It is conventionally regarded as a tumor suppressor because PP2A holoenzymes restrain PI3K–AKT–mTOR, RAS–RAF–MEK–ERK, MYC, Wnt/β-catenin, and cell-cycle signaling (Khanna et al., 2013; Sangodkar et al., 2017; Wlodarchak and Xing, 2016; Neale et al., 2025). PP2A is not a single functional entity. It is a modular family of heterotrimeric holoenzymes in which A scaffolding, B regulatory, and C catalytic subunits determine substrate specificity, subcellular localization, and biological role (Sontag et al., 2022; Wlodarchak and Xing, 2016). Consequently, distinct PP2A complexes can have different, and sometimes opposing, effects within the same signaling network (Arnold and Sears, 2006; Adams et al., 2005; Kuo et al., 2008; Lambrecht et al., 2018; Zhang et al., 2009; Johnson et al., 2024).

This holoenzyme diversity explains the apparent paradox of therapeutically inhibiting a tumor suppressor. Chronic PP2A dysfunction can facilitate malignant transformation by releasing oncogenic signaling, impairing genome-protective control, and promoting resistance to apoptosis (Khanna et al., 2013; Baldacchino et al., 2014; Neale et al., 2025). In contrast, established tumors frequently survive therapy by relying on residual phosphatase-dependent checkpoint adaptation, DNA-damage recovery, and stress-survival programs. A short, treatment-timed inhibitory pulse may impair these recovery processes, promote inappropriate mitotic entry in the presence of incompletely repaired DNA, and increase mitotic catastrophe or apoptosis (Hong et al., 2015; Chang et al., 2015; Zhang et al., 2015; D’Arcy et al., 2019; Chung et al., 2017). Thus, PP2A inhibition is not intended to produce sustained phosphatase loss; rather, it is intended to transiently remove a stress-adaptation buffer in tumor cells that are already close to a viability threshold.

The resulting vulnerability is expected to depend on tumor context. Possible determinants include high replication stress, chromosomal instability, defective homologous-recombination repair, and dependence on WEE1/CHK1/PLK1 or ATM/ATR signaling (Chang et al., 2015; Zhang et al., 2015; Hong et al., 2015; D’Arcy et al., 2019). *TP53* status may influence the balance between p53-dependent cell-cycle arrest and alternative outcomes following DNA damage, including aberrant mitotic entry and mitotic catastrophe, because PP2A participates in checkpoint and DNA-damage-response signaling (Yam et al., 2022; Qin et al., 2023; Hong et al., 2015; Lu et al., 2009). However, *TP53* status cannot be considered as a stand-alone predictor of response to PP2A inhibition.

LB100 is the leading clinical example of this PP2A inhibition strategy. It is a small-molecule catalytic inhibitor of PP2A and PP5 (protein phosphatase 5) that has primarily demonstrated preclinical chemo-, radio-, and immune-sensitizing activity, with early clinical feasibility established in a first-in-human dose-escalation study (Hong et al., 2015; Chung et al., 2017; D’Arcy et al., 2019; Ho et al., 2018; Dias et al., 2024b). Its translational rationale is therefore strongest when framed as schedule-specific, combination-based, and biomarker-informed: a short PP2A/PP5-directed inhibitory pulse is used to increase therapy-induced stress in a susceptible tumor while allowing recovery in normal tissues. This framework also makes safety central, because PP2A has essential physiological functions, particularly in the nervous system, where sustained dysfunction is associated with neurodevelopmental disorders and neurodegeneration (Verbinnen et al., 2021; Clark and Ohlmeyer, 2019; Sontag et al., 2004; Lee et al., 2011).

Mutations in PP2A subunits cause severe neurodevelopmental disorders. Moreover, it has been recently proposed that PP2A is a therapeutic target in inflammation and neurodegeneration (Clark and Ohlmeyer, 2019). Examination of postmortem Alzheimer’s disease (AD) patients’ brains revealed a reduced PP2A presence (Gong et al., 1993; Gong et al., 1995; Vogelsberg-Ragaglia et al., 2001; Sontag et al., 2004). PP2A activity is also decreased in brain regions with AD (Gong et al., 1993; Sontag et al., 2004; Javadpour et al., 2019). On the other hand, levels of endogenous PP2A inhibitors are increased (Tanimukai et al., 2005), which results in cardinal features of AD (Wang et al., 2010; Voronkov et al., 2011).

Since PP2A is involved in both tumor formation and dementia, this makes the task of treating cancer less trivial than it may seem at first glance. It is possible to assume that the action of a PP2A inhibitor such as LB100 can fight tumor development, and at the same time may, in principle, carry a risk of neurological side effects by disrupting PP2A in the brain.

This review therefore examines PP2A across its roles in the cell cycle, cancer, neurodevelopment and neurodegeneration, and evaluates the therapeutic rationale and safety limits of PP2A inhibition, with LB100 as the lead example.

The central translational question is whether transient PP2A inhibition can be confined to a therapeutic corridor that enhances tumor-cell vulnerability while preserving PP2A-dependent homeostasis in normal tissues, particularly the nervous system. The neurodevelopmental and neurodegenerative evidence therefore provides a biological framework for evaluating the neurological safety boundary of PP2A-targeted cancer therapy.

## 2 PP2A as a modular phosphatase system

Reversible phosphorylation of serine and threonine residues is one of the most widespread and important regulatory post-translational modifications determining the functional state of proteins. The human genome encodes roughly 3.5 times more kinases than phosphatases (656 vs. 184), meaning that each phosphatase complex must dephosphorylate a far broader range of substrates, which causes an imbalance and underscores the exceptional functional load carried by individual phosphatases such as PP2A (Smoly et al., 2017). The role of phosphatase enzymes has historically been studied far less thoroughly than that of kinases. Accumulating evidence confirms that the balance of phosphorylation of signaling molecules is key to regulating cell signaling in the nervous system. In this context, PP2A occupies a central position: it is the most highly expressed serine/threonine phosphatase in the brain and accounts for more than 50% of all Ser/Thr dephosphorylations in the cell (Verbinnen et al., 2021). By removing phosphate groups from a wide range of substrates in diverse tissues, PP2A performs key regulatory functions in cellular signaling and physiology, including the development and function of the nervous system.

PP2A works as a major controller of kinase-driven signaling; it can form many different heterotrimeric holoenzymes (60–96 in human), each assembled from a scaffolding A/PR65 subunit, one of four B regulatory subunit families (B/B55, B'/B56, B''/PR72, B‴/Striatin), and a catalytic C subunit (Sontag et al., 2022). B subunits are crucial for the broad substrate specificity of PP2A.

Between 50 and 100 different PP2A complexes can be present simultaneously in neurons, and the total number of identified substrates exceeds 300. PP2A regulates the activity of multiple signaling pathways in neurons, such as Wnt, mTOR, and MAPK, as well as the activity of numerous kinases responsible for the function and transport by neurotransmitter receptors, ion channels, transcription factors, and cytoskeletal structural elements. This makes PP2A a key regulator of neural transmission, homeostasis, and plasticity. Understanding the subunit-specific functions of PP2A provides key insights into the mechanisms of neuronal development, synaptic plasticity, and the pathogenesis of a wide range of neurological diseases, from neurodevelopmental syndromes to neurodegeneration.

The modular organization of PP2A is central to both its therapeutic tractability and its risk profile. Distinct A, B, and C subunit combinations determine substrate selection, subcellular localization, and tissue-specific biological effects; therefore, pharmacological PP2A modulation cannot be regarded as a uniform intervention. In tumors, interference with selected PP2A-dependent checkpoint and stress-recovery functions may create a conditional vulnerability, whereas in neurons, reduced PP2A activity may disrupt phosphoregulatory programs required for differentiation, synaptic maintenance, and proteostasis (Sontag et al., 2022; Verbinnen et al., 2021; Clark and Ohlmeyer, 2019).

## 3 PP2A in neurodevelopment and neuronal vulnerability

### 3.1 The role of PP2A in neuronal differentiation

The first indications of PP2A’s involvement in neuronal differentiation were obtained by (Strack, 2002): it was demonstrated that PP2A can act as a positive regulator of this process, with this function strictly dependent on the composition of its regulatory subunits. Thus, the PP2A–B56γ complex specifically dephosphorylates and activates Raf-1 kinase at the inhibitory residue Ser259, which enhances and prolongs signaling through the MAPK (MEK/ERK) cascade. As a result, PC12 cells exhibit a marked increase in NGF-induced neurite outgrowth. This study provides key evidence for a paradoxical pro-signaling role of a specific holoenzyme PP2A in neuronal development (Strack, 2002; Table 1). However, the role of PP2A in neuronal differentiation is ambiguous and is determined by the composition of its regulatory subunits. Contrary to expectations, knockout of individual subunits such as B56β or B56γ does not enhance, but rather blocks, differentiation, as it disrupts specific pro-neurogenic programs. This suggests that these holoenzymes perform a positive regulatory function: for example, they maintain TrkA activity (B56β/B56δ subunits) or relieve epigenetic repression (B56γ). Thus, PP2A acts in the differentiation process as both an activator and a suppressor, depending on the cellular context and subunit composition (Van Kanegan and Strack, 2009).

**TABLE 1:** Holoenzyme-specific PP2A functions: tumor-suppressive vs. context-dependent pro-signaling complexes.

Holoenzyme	Substrate/target	Functional outcome	Classification	References
PP2A–B56α/ε	AKT1 (Thr308, Ser473)	Restrains PI3K–AKT–mTOR signaling	Tumor-suppressive	Kuo et al., 2008; Nadel et al., 2024
PP2A–B56δ	c-Myc	Promotes c-Myc proteasomal degradation	Tumor-suppressive	Arnold and Sears, 2006
PP2A–B56/B56β	ERK1/2	Terminates MAPK signaling pulses	Tumor-suppressive	Adams et al., 2005; Lao et al., 2007
PP2A–B56γ	p53	Stabilizes p53, sustains p21-dependent arrest	Tumor-suppressive	Shouse et al., 2008
PP2A–B56δ	GSK-3β/β-catenin	Restricts Wnt-driven transcription	Tumor-suppressive	Lambrecht et al., 2018; Sanjari et al., 2020
PP2A–B'γ (B56γ)	Raf-1 (Ser259)	Activates MAPK/ERK, drives neurite outgrowth	Context-dependent pro-signaling (neuronal)	Strack, 2002
PP2A–B55α	β-catenin	Protects β-catenin from degradation, enables nuclear accumulation	Context-dependent pro-signaling	Panicker et al., 2020
PP2A–B55α	KSR1/Raf-1	Activates MEK1, drives MAPK signaling	Context-dependent pro-signaling	Johnson et al., 2024
PP2A–B‴/Striatin 3	MST1/2	Activates YAP/TAZ, suppresses Hippo pathway	Pro-oncogenic	Shor-Nareznoy et al., 2025; Tang et al., 2020

### 3.2 The role of PP2A in brain development

At the cellular level, the role of PP2A in the formation of neuronal architecture has been most thoroughly studied in relation to axons. Increased expression of the PP2A catalytic subunit (PP2Ac) not only stimulates the formation and elongation of functional axons but also restores their growth impaired by phosphatase inhibition. One of the key PP2A substrates in this context is the protein CRMP2 (collapsin response mediator protein 2), which is involved in axon polarization: dephosphorylation of CRMP2 is necessary for normal axonal growth, while constitutive expression of a phosphomimetic CRMP2 abolishes the effect of PP2A activation (Zhu et al., 2010). In addition, the specific *Ppp2r2c*/PP2A-B55 complex locally dephosphorylates key proteins in the axon initial segment (AIS), in particular the potassium channel Kv1.2 (Anderson et al., 2025). PP2A is also important for dendritic development. It functions as an effector of the transcriptional regulator Cut, which governs dendritic development by controlling cell-specific diversity and neuronal compartmentalization (Bhattacharjee et al., 2022). In *Drosophila* sensory neurons, PP2A is required for dendritic pruning and maintaining proper microtubule polarity (Rui et al., 2020). At the organismal level, the importance of PP2A is demonstrated by data from conditional knockouts. *Ppp2r2a*−/− embryos are significantly smaller than the wild type and have severe neurological defects such as exencephaly, spina bifida, and cranial vault collapse as well as syndactyly and severe epidermal defects (Panicker et al., 2020). PP2Acα mediates coordinated control of phosphorylation and protein homeostasis at the cellular and systemic levels, and is a key regulator of cerebellar development (Li et al., 2026). Mutations in *CUL4B* disrupt human cortical neurogenesis via PP2A-dependent inhibition of AKT and ERK (Ma et al., 2024).

### 3.3 PP2A in neurodevelopmental and neurodegenerative diseases

When the phosphorylation balance is disrupted due to mutations in the genes encoding PP2A subunits, this leads to severe neurodevelopmental disorders encompassing a group of diseases that are now collectively termed “PP2A syndromes” (Verbinnen et al., 2021). Mutations in *PPP2CA*, *PPP2R1A*, and *PPP2R5D* are causally associated with intellectual disability, autism spectrum disorders, bipolar disorder, and spinocerebellar ataxia (Borsotto et al., 2007; Houge et al., 2015; O’Hearn et al., 2015; Shang et al., 2016; Reynhout et al., 2019; Sandal et al., 2025; Verbinnen et al., 2025).

The *PPP2R1A* gene, encoding the Aα scaffolding subunit, causes Houge–Jansens syndrome type 2 when mutated. A systematic review of 60 patients from 16 studies identified 26 pathogenic variants, predominantly *de novo* heterozygous missense mutations clustering in HEAT repeats 5–7. Developmental delay and intellectual disability were present in 100% of patients for whom relevant data were available. Correlation analysis showed that the p. Met180Val/Thr variant is associated with macrocephaly, severe intellectual disability, and hypotonia without epilepsy, while variants at position Arg258 are associated with microcephaly (Lee et al., 2025). Mutations in *PPP2R5D*, which encodes the regulatory subunit B56δ (highly expressed in the brain), cause Houge–Jansens syndrome type 1 (also known as MRD35). The clinical picture includes mild to severe neurocognitive delay, speech impairment, hypotonia, macrocephaly, motor skill delays, and autism spectrum disorders. All known pathogenic variants are *de novo* missense mutations, most of which are recurrent and localized in the conserved acidic loop of B56δ, which is required for holoenzyme assembly. Notably, mutations in *PPP2R2B*, encoding the neuron-specific subunit of PP2A, represent another molecular cause of neurodevelopmental disorders: CAG repeat expansion in this gene causes spinocerebellar ataxia type 12 (SCA12), and *de novo* monoallelic missense variants are associated with intellectual disability and developmental delay (Sandal et al., 2025). Mutations in the catalytic subunit of *PPP2CA* are characterized by marked clinical heterogeneity: functional studies revealed complete dysfunction of PP2A in four patients with relatively mild intellectual disability (indicating haploinsufficiency), while ten other patients exhibited mutation-specific biochemical abnormalities such as reduced protein expression, altered binding to the A and B subunits, impaired phosphatase activity, and C-terminal methylation. Four variants appear to implement a dominant-negative mechanism, correlating with severe intellectual disability (Reynhout et al., 2019). Overall, genetic data suggest that PP2A syndromes represent a functionally convergent group of neurodevelopmental disorders united by disruption of specific PP2A-dependent substrate programs during critical periods of brain development. Taken together, these disorders indicate that the brain is highly sensitive to disruption of PP2A holoenzyme architecture across scaffolding, regulatory, and catalytic subunits. These genetic disorders provide a developmental safety context for pharmacological PP2A inhibition, whereas the potential vulnerability of the mature brain is illustrated by tau-centered neurodegenerative mechanisms in AD (Verbinnen et al., 2021; Reynhout et al., 2019; Sontag et al., 2004).

## 4 PP2A dysfunction in Alzheimer's disease: mechanisms and therapeutic implications

### 4.1 The role of PP2A in Alzheimer’s disease

PP2A is the predominant serine/threonine phosphatase in the brain and is responsible for the majority of tau phosphatase activity. Accumulating evidence suggests that PP2A dysfunction is a central event in the pathogenesis of AD and contributes to tau hyperphosphorylation, amyloidogenic signaling, synaptic dysfunction, and neurodegeneration.

PP2A suppression drives two hallmark AD pathologies simultaneously: hyperphosphorylation of tau (leading to neurofibrillary tangles) and increased phosphorylation of amyloid precursor protein (APP), which promotes β-amyloid (Aβ) production and disrupts synaptic function (Sontag and Sontag, 2014). Reduced PP2A methylation has also been shown to correlate with tau hyperphosphorylation in AD brain tissue (Zhou et al., 2008).

Evidence of impaired PP2A activity in AD was presented by (Sontag et al., 2004), who demonstrated a significant decrease in PP2A activity in the frontal and temporal cortex of the AD brain. Importantly, this decrease was associated with a selective loss of the tau-associated holoenzyme PP2A-ABαC, caused by decreased expression of the regulatory Bα subunit. Decreased ABαC levels were strongly correlated with neurofibrillary tangle (NFT) pathology and dystrophic neurites, but not with amyloid plaque burden, suggesting a direct link between PP2A dysfunction and tau pathology (Sontag et al., 2004). Consistent with these observations, Liu and Tian reviewed evidence suggesting that decreased PP2A activity is a common feature of AD and contributes to tau hyperphosphorylation, abnormal APP processing, and neuronal degeneration (Liu and Tian, 2009).

Current evidence suggests that PP2A dysfunction in AD is multifactorial and not the result of a single molecular defect. One important mechanism involves inhibitory phosphorylation of the PP2A catalytic subunit at Tyr307. Liu et al. demonstrated that phosphorylated PP2Ac-Tyr307 accumulates in vulnerable brain regions, including the entorhinal cortex and hippocampus, where it co-localizes with pretangles and mature neurofibrillary tangles. Increased Tyr307 phosphorylation correlated with Braak stage and tau phosphorylation, suggesting that post-translational inhibition of PP2A directly contributes to disease progression (Liu et al., 2008).

Endogenous PP2A inhibitors represent an additional level of regulation (Arif et al., 2014). demonstrated that cytoplasmic retention of I2PP2A/SET (endogenous PP2A inhibitor) suppresses PP2A activity, induces tau hyperphosphorylation, and promotes neurodegeneration. Importantly, I2PP2A-mediated PP2A inhibition was accompanied by activation of tau kinases, including GSK-3β and CaMKII, thereby enhancing pathological tau phosphorylation through multiple converging mechanisms (Arif et al., 2014).

The pathogenic significance of endogenous PP2A inhibition was further established by (Wang et al., 2015), who provided direct *in vivo* evidence that increased expression of I1PP2A is sufficient to induce an AD-like phenotype. Viral overexpression of I1PP2A in rat brain reduced PP2A activity, increased demethylated PP2Ac, promoted tau hyperphosphorylation, decreased synaptic markers, induced neurodegeneration and caused cognitive impairment. These findings established a causal sequence linking endogenous PP2A inhibition to tau pathology and cognitive dysfunction: I1PP2A elevation → PP2A inhibition → tau hyperphosphorylation → synaptic dysfunction → cognitive decline (Wang et al., 2015).

Importantly, PP2A regulates tau phosphorylation within a complex signaling network rather than through simple direct dephosphorylation (Qian et al., 2010). demonstrated that inhibition of PP2A produces site-specific changes in tau phosphorylation and simultaneously alters the activity of glycogen synthase kinase-3β (GSK-3β). Consequently, the final phospho-tau profile depends not only on loss of tau dephosphorylation but also on secondary effects on kinase signaling. PP2A therefore acts both directly on tau and indirectly through regulation of major tau kinases, highlighting its role as a central phosphoregulatory hub (Qian et al., 2010).

Additional complexity arises from the substrate-specific regulation of PP2A activity. Phosphorylation and dephosphorylation of tau regulate its physiological role in microtubule stabilization as well as its pathological aggregation into AD-associated fibrils in a highly dynamic and complex manner. The balance between multiple kinases and phosphatases determines the specific phosphorylation pattern of tau (Landrieu et al., 2011). demonstrated that the major tau phosphatase holoenzyme PP2A-PR55-Bα dephosphorylates different tau phosphorylation sites with varying efficiency. In particular, phosphorylation of tau at Thr231 was shown to impair PP2A-mediated dephosphorylation of pathological AT8 epitopes (Ser202/Thr205), whereas the prolyl isomerase Pin1 counteracted this inhibitory effect and facilitated tau dephosphorylation. These findings suggest that tau conformation itself can modulate PP2A activity and may contribute to the persistence of pathological tau hyperphosphorylation in AD (Landrieu et al., 2011).

PP2A stimulation shows strong therapeutic promise across several neurodegenerative diseases particularly AD and, more broadly, tauopathies and synucleinopathies, while reduced PP2A activity is a common driver of pathological protein hyperphosphorylation and aggregation in these disorders (Javadpour et al., 2019).

Because PP2A dysfunction occupies a central position at the intersection of tau pathology, amyloidogenic processing and synaptic signaling, restoration of PP2A activity has emerged as an attractive therapeutic strategy (Voronkov et al., 2011). proposed several approaches to PP2A-targeted therapy, including direct allosteric activation of PP2A, disruption of PP2A interactions with endogenous inhibitors such as SET/I2PP2A, and modulation of post-translational modifications that control holoenzyme assembly and activity. The authors argued that PP2A activation might offer advantages over selective kinase inhibition because PP2A simultaneously regulates multiple pathogenic pathways involved in AD (Voronkov et al., 2011).

Pharmacological PP2A activators, such as the synthetic tricyclic sulfonamide compounds known as small-molecule activator of PP2A (SMAPs), increase PP2A activity and have demonstrated clear disease-modifying effects in preclinical AD models (Wei et al., 2020). In cell models (HEK293/tau and N2a/APP lines), SMAPs reduced tau phosphorylation and lowered Aβ40/42 levels. In hyperhomocysteinemia-induced AD rat models, SMAP treatment rescued cognitive impairments, reduced tau hyperphosphorylation and Aβ overproduction, and prevented neuronal dendritic spine loss and synaptic impairment in the hippocampus (Wei et al., 2020). Therefore, pharmacological reactivation of PP2A shows promise as a therapeutic strategy for AD, which is typically treated only symptomatically (Voronkov et al., 2011).

Taken together, these data support the view that PP2A dysfunction is not simply a consequence of AD pathology, but a mechanistically significant and potentially reversible signaling node linking tau hyperphosphorylation, amyloidogenic signaling, synaptic failure, and neurodegeneration.

### 4.2 Consequences of PP2A targeting on neurodegeneration

PP2A inhibition is most extensively studied using the natural toxin okadaic acid (OKA) as a pharmacological tool (Kamat et al., 2014). OKA is a potent and selective inhibitor of PP1 (protein phosphatase 1) and PP2A that reliably induces tau hyperphosphorylation and AD-like pathology in both *in vitro* and *in vivo* models (Kamat et al., 2014). Microinfusion of OKA into rat brain produces cognitive deficits, neurofibrillary tangle-like changes, and oxidative stress, mirroring the tau-driven pathology seen in AD patients (Kamat et al., 2013).

PP2A also dephosphorylates α-synuclein at serine 129. Impaired PP2A activity is linked to increased α-synuclein phosphorylation and aggregation, both pathogenic features of Parkinson’s disease (PD) (Lee et al., 2011). Conversely, boosting PP2A methylation and activity in α-synuclein transgenic mice markedly reduces serine 129 phosphorylation and aggregation, reinforcing that insufficient PP2A activity leads to synucleinopathy progression (Lee et al., 2011).

Endothall, a herbicide structurally related to cantharidin, has been shown to inhibit PP2A activity in neuronal and other cell models, producing neurotoxic effects (Li and Casida, 1992). Cantharidin analogues, including endothall, bind to the same PP2A site in brain and liver, and their cytotoxic potency correlates with displacement of radioligand binding at this site (Graziano and Casida, 1987).

LB100 was rationally engineered as a watersoluble PP2A inhibitor derived from norcantharidin, specifically designed to reduce the human toxicities associated with earlier PP2A inhibiting compounds (Hong et al., 2015; Scott et al., 2026). A first-in-human Phase 0 “window of opportunity” trial (NCT03027388) was conducted to determine whether LB100 crosses the human blood–brain barrier (BBB) and to characterize its pharmacokinetics in tumor versus plasma tissue (Burton et al., 2025). This BBB penetration question is clinically important because a PP2A inhibitor reaching brain tissue potentially could trigger downstream tau hyperphosphorylation and autophagy disruption.

However, LB100 achieved only 0.31% average brain tumor penetration relative to plasma concentration roughly 1,000-fold below the drug’s PP2A inhibitory IC50 (half-maximal inhibitory concentration), demonstrating that *intact* LB100 crosses the BBB too poorly to achieve pharmacologically relevant PP2A inhibition (Burton et al., 2025).

These data suggest limited direct central nervous system (CNS) target engagement by intact LB100 under the study conditions, although they do not establish the distribution or pharmacological activity of endothall in normal brain tissue.

### 4.3 From neuronal vulnerability to anticancer targeting: defining the therapeutic corridor

The evidence reviewed above indicates that PP2A inhibition is not biologically neutral in the nervous system. Disruption of PP2A subunits causes severe neurodevelopmental phenotypes, whereas reduced PP2A activity in the mature brain is associated with tau hyperphosphorylation, α-synuclein pathology, synaptic dysfunction, and neurodegeneration (Verbinnen et al., 2021; Sontag et al., 2004; Lee et al., 2011; Wang et al., 2015). These observations justify careful assessment of CNS exposure and treatment duration for PP2A-directed cancer therapies, particularly when inhibition is sustained or when BBB integrity may be compromised.

Conversely, the broad regulatory role of PP2A can create a therapeutic vulnerability in established cancer cells. Tumors frequently experience persistent oncogenic, replicative, proteotoxic, and genotoxic stress and may retain dependence on PP2A/PP5-linked checkpoint adaptation and recovery pathways. In this setting, a transient phosphatase-inhibitory pulse may impair recovery from therapy-induced damage and promote aberrant mitotic entry or mitotic catastrophe, particularly in combination with cytotoxic therapy, radiotherapy, or immunotherapy (Hong et al., 2015; Chung et al., 2017; D’Arcy et al., 2019; Ho et al., 2018).

A favorable therapeutic corridor would require pharmacologically meaningful PP2A/PP5-directed modulation in tumor tissue during therapy-induced stress while keeping exposure in the CNS and other normal tissues below the threshold for persistent disruption of PP2A-dependent homeostatic programs. Relevant determinants should include systemic exposure to both LB100 and endothall; tumor-to-plasma and brain-to-plasma partitioning; BBB penetration; the magnitude and duration of target inhibition; tumor dependence on PP2A/PP5-mediated recovery pathways; and use of intermittent rather than continuous dosing schedules (Chung et al., 2017; D’Arcy et al., 2019; Feng et al., 2023; Rollema et al., 2025; Burton et al., 2025).

## 5 PP2A role in cancer

### 5.1 Differences between PP2A normal vs. malignant functioning

In non-transformed cells, PP2A constrains major oncogenic pathways and contributes to DNA-damage checkpoint control. In established tumors, however, persistent signaling stress, genomic instability, and partial disruption of PP2A regulation may create dependence on residual PP2A/PP5-mediated recovery functions. This context dependence explains why chronic loss of PP2A activity can promote tumorigenesis, whereas transient and treatment-timed inhibition may nevertheless sensitize selected tumors to anticancer therapy (Sarais et al., 2025; Fujiki et al., 1991; Hong et al., 2015; D’Arcy et al., 2019).

In cancer cells, PP2A balanced phosphatase activity is compromised. The conversion of PP2A from tumor suppressor to oncogene happens through several convergent mechanisms: somatic mutations in its subunits, transcriptional and post-translational suppression of regulatory subunits, overexpression of endogenous PP2A inhibitor proteins, and post-translational modification of the catalytic subunit (Khanna et al., 2013). The result of PP2A transition is constitutive phosphorylation of growth-promoting PP2A substrates driving sustained proliferative signaling, resistance to apoptosis, replicative immortality, and therapy resistance (Neale et al., 2025).

#### 5.1.1 Mechanisms of PP2A dysregulation in cancer

The most extensively documented genetic mechanism of PP2A inactivation is somatic mutation of the *PPP2R1A* scaffolding subunit, which carries the highest frequency of cancer-associated mutations in different tumor types. These mutations influence the B-subunit binding (heat repeat regions HR5 and HR7), with hotspot substitutions R183W/Q, P179R, R182W, and W257C that were reported in uterine serous carcinoma, ovarian clear-cell carcinoma, melanoma, lung, and breast cancers (Calin et al., 2000; Shih et al., 2011). These mutations generate a dominant-negative PP2A holoenzyme with impaired B-subunit docking, while the A–C dimer can still bind substrates but cannot dephosphorylate them (Sarais et al., 2025). The biological potency of this mechanism is demonstrated by the E64D *PPP2R1A* knock-in mouse, which develops 50% more lung cancers after carcinogen exposure, showing that the RAS/MAPK axis is a primary target whose controlling requires intact PP2A-A subunit (Walter and Ruediger, 2012). Mutations in *PPP2R1B* also occur in colorectal, breast, and lung carcinomas, disrupting PP2A-C binding (Wang et al., 1998; Baldacchino et al., 2014). Deletions of *PPP2R2A* (encoding B55α) were shown in breast and prostate cancer (Beca et al., 2015; Zhao et al., 2019), while approximately 70% of cancer patients harbor heterozygous deletions or missense mutations in *PPP2R4/PTPA*, the cellular PP2A activator, reducing its ability to activate the catalytic subunit (Sents et al., 2017).

PP2A could be also inactivated through overexpression of endogenous inhibitor proteins such as SET (also known as I2PP2A or TAF-Iβ) and CIP2A (Ribaudo et al., 2025; Figure 1). SET directly binds and occludes the PP2A catalytic subunit’s active site, and its overexpression is a defining feature of chronic lymphocytic leukaemia (CLL) and acute myeloid leukaemia (AML), as well as being found in colorectal and breast cancers (Ribaudo et al., 2025). CIP2A achieves PP2A inhibition through a distinct mechanism. It stabilises c-Myc by blocking PP2A–B56α-mediated Ser62 dephosphorylation and prevents c-Myc’s proteasomal degradation, while its expression correlates with poor prognosis across multiple solid tumours (Pavic et al., 2023). In-silico analysis of basal breast cancer showed that almost 60% of tumours harboured PP2A deregulation, many with subunit loss or inhibitory subunit upregulation and some with elevated CIP2A or SET, illustrating that different mechanisms overlap and contribute to the PP2A inhibition (Baldacchino et al., 2014). Another mechanism of inactivation involves post-translational modification of PP2A-C. Phosphorylation of the catalytic subunit’s Y307 residue, methylation status changes at L309 (regulated by LCMT-1 (leucine carboxymethyltransferase 1) and PME-1 (protein phosphatase methylesterase 1) affecting B55 holoenzyme stability (Johnson et al., 2024). Phosphorylation at T304 by CDK1 during mitosis that displaces B55 regulatory subunits (Nasa et al., 2020). All these mechanisms contribute to context-dependent PP2A inactivation in tumor cells.

**FIGURE 1:**
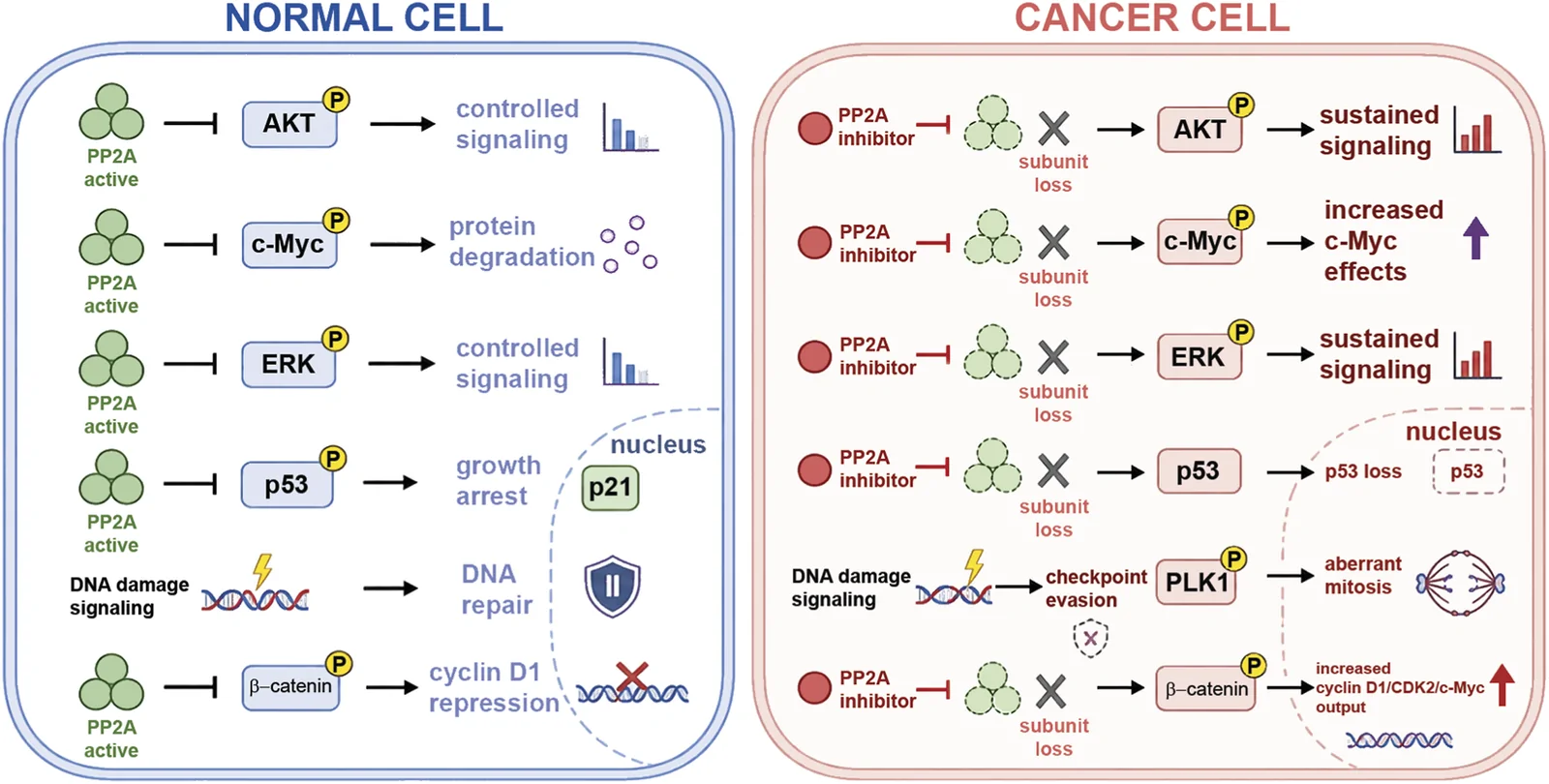
PP2A-dependent signaling in normal versus cancer cells. Schematic comparison of PP2A tumor-suppressor signaling in a normal cell (left) and a cancer cell with pharmacological or genetic PP2A inactivation (right). Normal cell: Active PP2A holoenzyme (green trimer) dephosphorylates AKT, c-Myc, ERK, p53, and β-catenin, keeping proliferative and survival signaling under control, promoting c-Myc degradation, and repressing Cyclin D1. Upon DNA damage, PP2A-dependent p53 activation induces p21 and G1/S arrest, allowing DNA repair and preservation of genome integrity. Cancer cell: A PP2A inhibitor (red circle) binds the holoenzyme and causes loss of a regulatory subunit (X), abolishing phosphatase activity. Loss of PP2A activity results in constitutive phosphorylation and sustained activity of AKT and ERK, increased c-Myc output, and loss of functional p53. DNA damage signaling no longer triggers cell-cycle arrest; instead, checkpoint evasion permits continued PLK1 activity and aberrant mitosis. Concurrent loss of β-catenin regulation increases Cyclin D1, CDK2, and c-Myc output, driving unchecked proliferation. Symbols: Green trimer, active PP2A holoenzyme; pale dashed trimer, inactive/subunit-depleted PP2A; red circle, PP2A inhibitor; yellow “P,” phosphorylation; blunt-ended line, inhibition; pointed arrow, activation; “X,” loss of a PP2A regulatory subunit; lightning bolt, DNA damage signal.

#### 5.1.2 PP2A role in cell cycle

PP2A functions as an important regulator of key transitions in the cell cycle, with diverse holoenzymes governing decisions about whether a cell proceeds, pauses, or exits the cycle. It can dephosphorylate many substrates involved in the cell cycle through diverse holoenzyme combinations. In this way, PP2A affects pathways such as Wnt, mTOR, and MAPK, as well as critical processes like the G1-to-S transition, DNA synthesis, and the initiation of mitosis (Wlodarchak and Xing, 2016).

During the G1 phase, the PP2A–B55α dephosphorylates β-catenin and promotes Wnt-driven signaling leading to the expression of cyclin D1 and c-Myc, and facilitating the assembly of CDK4/6 (Panicker et al., 2020; Table 1). In contrast, the PP2A–B56α dephosphorylates c-Myc, enhancing its degradation, while PP2A–B56δ regulation of the GSK3β axis also contributes to destabilizing c-Myc (Arnold and Sears, 2006; Figure 1; Table 1). Therefore, PP2A role is to fine-tune the cell’s entry into the cycle rather than completely blocking it (Wlodarchak and Xing, 2016).

As cells enter S phase, PP2A counterbalances CDK9 within the PTEFb complex to ensure that RNA polymerase II completes transcripts correctly, preventing premature termination and loss of poly(A) tails (Tellier et al., 2022). This regulation supports the expression of E2F-driven cyclins E and A, which are necessary for S-phase commitment. PP2A also influences S-phase progression through DNA replication and checkpoint signaling (Wlodarchak and Xing, 2016).

In the G2/M checkpoint, PP2A helps control PLK1 activity in the presence of unrepaired DNA damage (Figure 1), functioning within the broader ATM/ATR–CHK1/2–CDC25 signaling network that prevents premature entry into mitosis and maintains p53-dependent responses to DNA damage (Yam et al., 2022).

The best-studied role of PP2A in the cell cycle occurs at the transition to mitosis. During late G2 and early mitosis, cyclin B–CDK1 phosphorylates PP2A-C at Thr304, disrupting the formation of PP2A-B55 holoenzymes and increasing phosphorylation of CDK1 substrates. This promotes mitotic entry (Nasa et al., 2020). When cyclins A and B are degraded by APC/C at the metaphase-to-anaphase transition, mitotic entry is abolished with the reactivation of PP2A–B55 (Nasa et al., 2020). PP2A–B55α then dephosphorylates CDK1 substrates, facilitating mitotic exit by ensuring proper spindle localization, chromosome decondensation, nuclear envelope reformation, and cytokinesis (Lacroix et al., 2022).

#### 5.1.3 PP2A in oncogenic signaling

The oncogenic implications of PP2A inactivation depend on which pathways the major holoenzymes normally suppress and which changes occur when that suppression fails.

Concerning the PI3K–AKT–mTOR pathway, PP2A–B56α/ε dephosphorylates AKT1 at Thr308 and Ser473 as well as mTOR and therefore maintains balanced growth signaling in normal non-transformed cells (Kuo et al., 2008; Nadel et al., 2024; Figure 1; Table 1). PP2A–B56 and PP2A–B55 dephosphorylate the downstream effectors of PI3K–AKT–mTOR pathway, S6K and 4E-BP1, thus inhibiting cap-dependent translation and ribosomal biogenesis (Zhang et al., 2024; Lum et al., 2025). In cancer, loss of these two holoenzymes unlocks constitutive AKT–mTOR activity and contributes to resistance to PI3K inhibitors, because the pathway can no longer be fully suppressed by upstream blockade due to inappropriate PP2A activity. Furthermore, active mTOR inhibits PP2A, while the mTOR regulator TIPRL binds to PP2A-C to stimulate mTORC1. This creates a loop in which loss of PP2A activity amplifies mTOR activity (Scorsato et al., 2016).

The influence on MAPK/ERK pathway is achieved by PP2A–B56/B56β which directly dephosphorylates ERK1/2 (Figure 1). Dephosphorylation of Sprouty2 disrupts upstream RAS activation (Adams et al., 2005; Lao et al., 2007; Table 1) and limits transcription of Elk1, Fos, and Jun target genes (Liu et al., 2013; 2016; Wang et al., 2022). The simultaneous occurrence of PP2A mutations with KRAS mutations removes ERK suppression from two distinct sides and allows it to amplify oncogenic signaling (Sangodkar et al., 2017). In contrast, PP2A-B55α dephosphorylates kinase suppressor of Ras 1 and Raf-1, which activates MEK1 and drives MAPK signaling (Johnson et al., 2024; Table 1), thus illustrating that PP2A’s effects on MAPK are not uniformly inhibitory. The preferential outcome in any given cancer type depends on which holoenzyme is lost and which is retained.

Similarly, PP2A shows bidirectional regulation of the Wnt/β-catenin pathway. PP2A–B56δ activates GSK-3β by dephosphorylation, which in turn phosphorylates β-catenin and marks it for ubiquitin-mediated proteasomal degradation, thus suppressing Wnt-driven transcription of cyclin D1 and c-Myc (Lambrecht et al., 2018; Sanjari et al., 2020; Figure 1; Table 1). In contrast, PP2A–B55 dephosphorylates β-catenin, protecting it from degradation and enabling its nuclear accumulation (Zhang et al., 2009). In cancers where B56 is lost but B55 activity is preserved, PP2A’s role shifts from tumor-suppressive to tumor-tolerant within the MAPK/ERK and Wnt/β-catenin pathways.

PP2A also regulates the p53-dependent DNA-damage response in a holoenzyme-specific manner. Serine 15 phosphorylation of p53 following genotoxic stress promotes its association with the B56γ regulatory subunit, and this B56γ-specific PP2A holoenzyme dephosphorylates and stabilizes p53, reinforcing p21-dependent cell-cycle arrest and tumor-suppressive activity (Shouse et al., 2008; Figure 1; Table 1). Loss or downregulation of B56γ can therefore compromise p53 stabilization and apoptotic checkpoint fidelity in cancer cells, consistent with the p53 destabilization observed when PP2A–B56 function is disrupted.

In the Hippo/YAP pathway, a specific PP2A isoform plays an entirely pro-oncogenic role: the B‴/Striatin 3 subunit recruits MST1/2 kinases and promotes their dephosphorylation, which activates YAP/TAZ and turns off Hippo-mediated growth suppression (Shor-Nareznoy et al., 2025; Table 1). Striatin 3 upregulation is associated with poor prognosis in gastric cancer (Tang et al., 2020). Therefore, functioning of Striatin 3 as an oncogenic facilitator should be considered when planning therapeutic strategies involving PP2A modulation.

### 5.2 Therapeutic targeting of PP2A in cancer

PP2A can be therapeutically targeted in two opposite ways: activation or inhibition. Both approaches can suppress tumor growth through distinct mechanisms and both strategies share the same clinical goal: selectively killing cancer cells while sparing normal tissue. Current development of PP2A targeting favors combination approaches rather than monotherapy in either direction (Mazhar et al., 2019).

Table 2 illustrates that PP2A activation and inhibition pursue distinct therapeutic objectives and are associated with different context-dependent limitations. PP2A activation aims to restore tumor-suppressive phosphatase functions, whereas transient PP2A inhibition is intended to sensitize treatment-stressed tumor cells by disrupting checkpoint and recovery pathways. For the latter strategy, toxicity in normal proliferating tissues and possible impairment of genomic integrity remain mechanistically possible, exposure-dependent concerns rather than established clinical consequences.

**TABLE 2:** Strengths and limitations of PP2A targeting approaches for cancer treatment.

Aspect	PP2A stimulation	PP2A inhibition
Core mechanism	Restores tumor-suppressor dephosphorylation of c-Myc, AKT, ERK (Raman and Pervaiz, 2019)	Disables checkpoint/DDR, forcing mitotic catastrophe (Lu et al., 2009)
Best-suited context	MYC-driven or oncogene-addicted tumors (Pippa and Odero, 2020)	Combination with chemo/radio/immunotherapy in tumors with unstable genome (Ho et al., 2018)
Key advantage	Restorative mechanism; spares normal hematopoiesis in AML models (Voronkov et al., 2026)	Strong chemo- and immune-sensitizing synergy; clinical-stage compounds (LB100) (Mazhar et al., 2019)
Key limitation	Context-dependent efficacy across cell types (Tinsley and Allen-Petersen, 2022)	Limited tumor selectivity of checkpoint/DDR perturbation; potential toxicity in normal proliferating tissues and endothall-related systemic exposure require schedule- and exposure-dependent evaluation (Mazhar et al., 2019; Chung et al., 2017; Feng et al., 2023; Rollema et al., 2025; Dias et al., 2025)
Systemic risk	Cardiovascular homeostasis disruption via β-adrenergic signaling (Vasudevan et al., 2011)	Potential impairment of genomic integrity in normal proliferating tissues during prolonged or excessive checkpoint/DDR inhibition (Ronk et al., 2022; Mazhar et al., 2019)

Small molecule activators of PP2A are re-engineered phenothiazine analogues, of which DT-061 is the best-characterized (Manoharan et al., 2022). DT-061 binds at the interface of all three PP2A subunits, and selectively stabilizes the PP2A–B56α holoenzyme in its active conformation, thus enabling substrate-specific dephosphorylation of c-Myc at Ser62 and AKT1 at Thr308 (Shah et al., 2020). In non-small cell lung carcinoma with KRAS mutations, DT-061 overcomes TKI (tyrosine kinase inhibitor) resistance when combined with afatinib (Tohmé et al., 2019), while in venetoclax-resistant chronic lymphoid leukemia, DT-061 induces apoptosis independently of Bax/Bak (Jayappa et al., 2023). It also decreases RAD51 expression to sensitize homologous recombination proficient high-grade serous carcinoma to PARP inhibitors (Avelar et al., 2023). In glioblastoma, in combination with AKT and PDK inhibitors, DT-061 inhibits mitochondrial OXPHOS and induces apoptosis (Denisova et al., 2023).​

Indirect activation strategies target the endogenous PP2A inhibitors themselves, SET and CIP2A. FTY720 (Fingolimod), the FDA-approved multiple sclerosis drug, binds SET, which is an endogenous PP2A inhibitor, blocking the SET–PP2A-C interaction and driving SET nuclear translocation away from cytoplasmic PP2A (Qadri et al., 2021). FTY720 was able to restore PP2A-mediated AKT and ERK dephosphorylation across colorectal, gastric, prostate, breast, lung, and ovarian cancer cells, neuroblastoma, and leukaemia models (Matsuoka et al., 2003; Li et al., 2013; Shrestha et al., 2018; Pournajaf et al., 2022). However, its clinical application in oncology is limited by Sphingosine1phosphate (S1P) receptor-mediated cardiotoxicity and lymphopenia (Bravo et al., 2022). Therefore, FTY720 analogues have been developed: OSU-2S targets SET without S1P receptor engagement, while CM-1231 eliminates cardiac toxicity as demonstrated in a zebrafish model and shows superiority to FTY720 in acute myeloid leukemia (Verrills, 2022). CIP2A suppression is achieved mainly through transcriptional downregulation. TD52, an erlotinib analogue, prevents ELK1 binding to the CIP2A promoter in triple-negative breast cancer and hepatocellular carcinoma (Yu et al., 2014; Liu et al., 2017). Bortezomib, a proteasome inhibitor, decreases CIP2A mRNA in colon and triple-negative breast cancer (Tseng et al., 2012; Ding et al., 2014). Celastrol promotes proteasomal degradation of CIP2A, leading to restored PP2A activity and suppressed cMycmediated oncogenesis in hepatocellular carcinoma, triple-negative breast cancer, and pancreatic ductal adenocarcinoma (Wang et al., 2023).

However, the pro-tumor-suppressor logic of PP2A stimulation has an important limitation (Table 2). Namely, PP2A’s composition and substrate specificity are highly context-dependent, and therefore, PP2A activating compounds do not uniformly suppress oncogenic signaling because their epigenetic and transcriptional effects may deviate considerably between cancer cell types and non-transformed cells (Tinsley and Allen-Petersen, 2022). PP2A also has essential physiological roles beyond oncology, as it regulates β-adrenergic receptor resensitization in the heart. Therefore, systemic PP2A activation risks disrupting homeostatic circuits in the organism (Vasudevan et al., 2011).

The alternative, and more extensively tested, strategy is pharmacological inhibition of PP2A and related phosphatases. The apparent contradiction between PP2A’s tumor-suppressive role and the use of its inhibitors is resolved by considering the timing and cellular context of intervention. Persistent PP2A dysfunction can promote tumorigenesis by releasing oncogenic signaling and weakening genome-protective control. In contrast, established tumors exposed to chemotherapy, radiotherapy, or other therapeutic stresses may rely on residual PP2A and other phosphatases activity to coordinate checkpoint adaptation, DNA-damage recovery, and stress-induced survival. Under these conditions, a short and treatment-timed inhibitory pulse may prevent appropriate recovery, promote aberrant mitotic entry in the presence of unresolved DNA lesions, and increase mitotic catastrophe or apoptosis. Thus, pharmacological PP2A inhibition is best considered a transient stress-sensitizing strategy in combination regimens, rather than a strategy for sustained suppression of a tumor suppressor (Hong et al., 2015; Chung et al., 2017; D’Arcy et al., 2019; Lu et al., 2009; Mazhar et al., 2019). Several small molecules established this concept experimentally.

Okadaic acid became a classic PP1/PP2A inhibitor but also revealed the toxicological limits of broad phosphatase suppression (Bialojan and Takai, 1988; Nigro et al., 1989). Fostriecin was studied as a natural antitumor agent with marked PP2A inhibitory activity (Bender et al., 1998). Cantharidin induced G2/M arrest and apoptosis in cancer cells, while norcantharidin was developed as a synthetic multi-target anticancer agent (He et al., 2020). Toxicity and poor target selectivity, however, constrained clinical development of this entire class, setting the stage for a more selective successor: LB100.

This historical context is essential for understanding LB100’s rationale, which centers on short-term interference with PP2A/PP5-dependent recovery programs that tumor cells rely on after therapeutic stress. Weakening the DNA damage response, checkpoint adaptation, and stress-induced survival pathways in this way can increase tumor sensitivity to cytotoxic or radiation treatment (Hong et al., 2015; Chung et al., 2017; D’Arcy et al., 2019). A possible immune-mediated component of this sensitization, linked to LB100-induced alternative splicing and immunopeptidome remodeling, is discussed later (Dias et al., 2024b).

PP5, unlike other PPP-family members, functions as a single-subunit enzyme, and its relevance to the LB100 rationale lies specifically in the DNA damage response. Following genotoxic stress, PP5 associates with and is required for activation of the apical checkpoint kinases ATM and ATR, dephosphorylates DNA-dependent protein kinase at Thr2609 to tune repair signaling, and directly dephosphorylates p53, which in turn represses PP5 transcription (Qin et al., 2023). Both PP5 and PP2A play important roles in the DNA damage response. When a compound inhibits both of these proteins, it weakens the response from two angles simultaneously. LB100 initially demonstrated selectivity for PP2A. It was later found that the phosphopeptide used to demonstrate this selectivity is also a substrate for PPP5C (D’Arcy et al., 2019). As a result, the dual inhibition of PP2A and PP5 by LB100 is believed to contribute additively to its antitumor activity.

This dual-target profile makes pharmacological PP2A/PP5 inhibition a strategy that requires a narrow therapeutic rationale, given that PP2A is broadly tumor-suppressive and restrains oncogenic signaling in most cancer contexts (Orimo et al., 2005; Xuan et al., 2013), while the same suppressive activity tumors already subvert endogenously via CIP2A, SET, and PME-1 (Carbia-Nagashima et al., 2007; Bernhagen, 2019). The approach is therefore best framed not as durable pathway blockade but as temporary sensitization: a short phosphatase-inhibitory pulse designed to impair tumor-cell recovery immediately after therapy. Because PP2A and PP5 are intracellular targets, antibody-based inhibitors are not a viable modality (therapeutic antibodies act on extracellular or membrane targets, and intracellular engagement would require specialized intrabody or delivery strategies), leaving small molecules as the only realistic option for testing this concept.

LB100 is the most clinically advanced molecule for this purpose. It is a water-soluble, cantharidin-derived small molecule that has progressed from preclinical combination studies to first-in-human testing with published dosing and early tolerability data in patients with advanced solid tumors (Hong et al., 2015; Chung et al., 2017).

LB100 prevents PP2A-mediated checkpoint execution at G2/M through loss of ATM/ATR-mediated DNA damage response leading to hyperphosphorylation of PLK1 and CHK1/CHK2 thereby forcing DNA-damaged cancer cells into aberrant mitosis and mitotic catastrophe (Hong et al., 2015; Scott et al., 2026). This inhibition strategy is paradoxical, since PP2A is itself a tumor suppressor, but it works by pushing an already genomically unstable cancer cell past a viability threshold rather than restoring normal control (Lu et al., 2009; Ronk et al., 2022). PP2A inhibitors including LB100 also arrest cells at G2/M through JNK/Sp1-dependent CDK1 downregulation and autophagy-dependent p21 upregulation, reinforcing this mitotic-catastrophe pathway (Gong et al., 2015).

LB100 has shown significant potential as a chemo- and radio-sensitizer. It synergizes with temozolomide in glioblastoma by activating AKT1, upregulating Plk-1, and decreasing p53 (Hong et al., 2015). In ovarian carcinoma and osteosarcoma, LB100 enhances cisplatin effects by inhibiting homologous recombination repair, which leads to abolishing S-phase arrest (Chang et al., 2015; Zhang et al., 2015).

Additional preclinical mechanisms include increased dependence on compensatory WEE1/CHK1 pathways, modulation of P-glycoprotein-associated drug resistance, and RNA-splicing changes with potential immunogenic consequences ((Gordon et al., 2015; Dias et al., 2024a; Dias et al., 2024b; Podolski-Renić et al., 2025). The latter opens an immunotherapy angle unavailable to PP2A activators, as LB100 combined with anti-PD-1 blockade synergistically produces durable immune-mediated antitumor responses (Ho et al., 2018). These mechanisms, summarized in Figure 2, support LB100’s combination-oriented positioning but remain context-dependent and do not yet define a single validated model of action.

**FIGURE 2:**
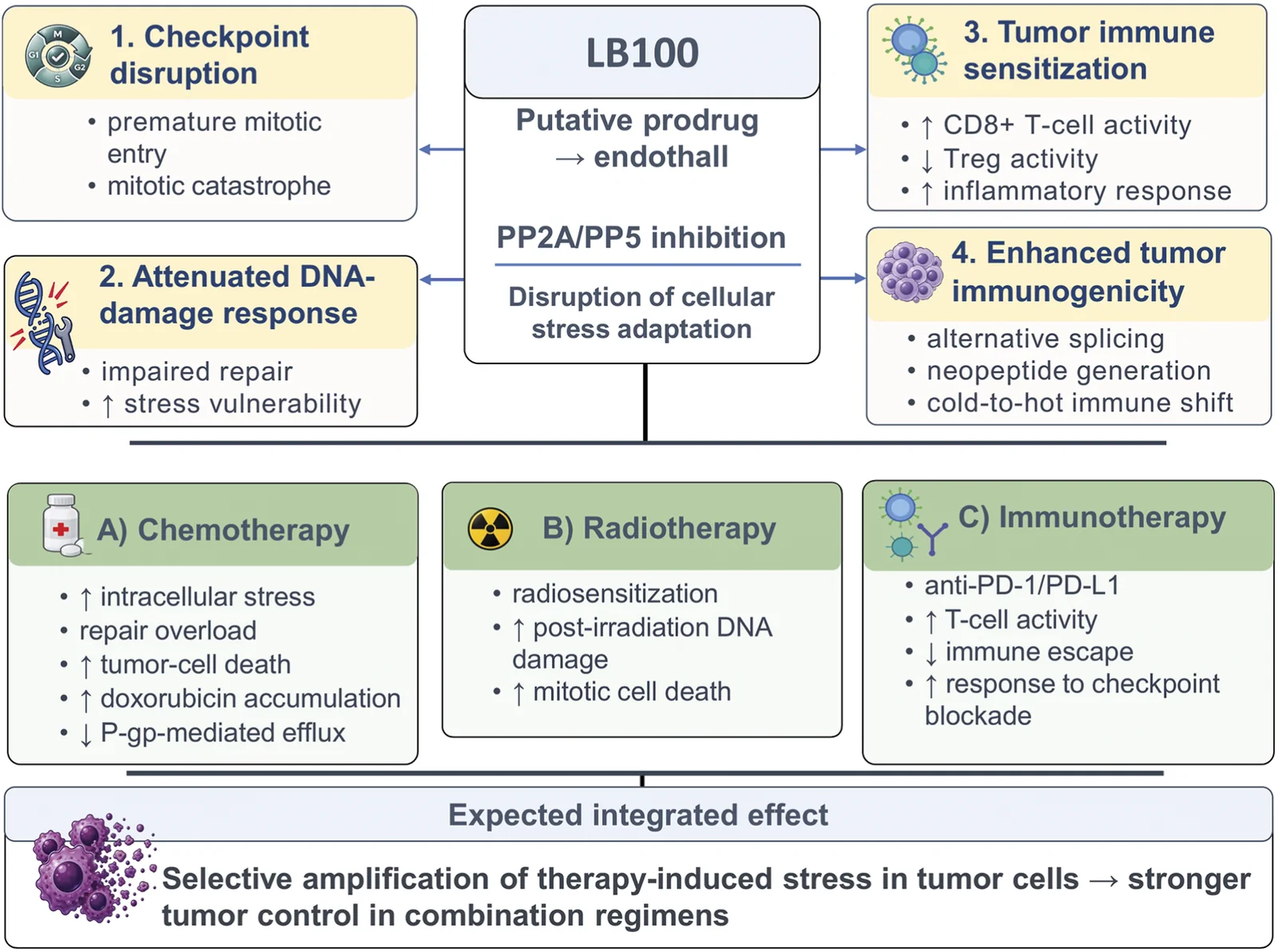
Proposed role of LB100 in combination anticancer therapy. LB100 is shown as a PP2A/PP5-directed small molecule that may undergo prodrug-like conversion to the pharmacologically active metabolite endothall and transiently disrupt tumor-cell stress adaptation. The scheme summarizes four context-dependent mechanisms that may contribute to therapy sensitization: checkpoint disruption with premature mitotic entry and mitotic catastrophe; attenuation of the DNA-damage response and repair; tumor immune sensitization; and enhanced tumor immunogenicity associated with alternative splicing and neopeptide generation. These mechanisms may be exploited in combination with chemotherapy, radiotherapy, or immunotherapy. Examples of chemotherapeutic agents investigated in combination with LB100 include temozolomide, cisplatin, and doxorubicin; P-glycoprotein-associated modulation of multidrug resistance may additionally enhance intracellular drug exposure in selected contexts. The expected integrated effect is increased therapy-induced stress and tumor sensitization, leading to stronger tumor control in combination regimens. The depicted mechanisms are context-dependent and remain predominantly preclinical; their magnitude and clinical relevance require tumor- and regimen-specific validation.

The cost of this strategy is that PP2A inhibition is fundamentally non-selective for malignant versus normal cells (Table 2), since it disables a checkpoint mechanism that healthy dividing cells also rely on for genomic fidelity (Mazhar et al., 2019). This is worsened in LB100’s specific case by its prodrug conversion to endothall, a caustic environmental toxicant, which constrains the therapeutic window and dosing regimen achievable in the clinic (Dias et al., 2025).

LB100 undergoes pH-dependent hydrolysis in aqueous solution and *in vivo* thereby releasing its active metabolite, endothall, which is a potent PP1/PP2A inhibitor and is responsible for prolonging phosphatase inhibition beyond the pharmacokinetic half-life of the parent compound (Feng et al., 2023; Rollema et al., 2025). Stability studies show LB100 degrades rapidly and only about 14% LB100 remains after 8 h in water, while it is stable under basic conditions. Therefore, pH determines the ratio of LB100 to active metabolite endothall that reaches systemic circulation (Rollema et al., 2025). A validated LC–MS/MS method developed to simultaneously quantify LB100 and endothall in human plasma confirmed that circulating endothall is generated during infusion and accumulates with repeated LB100 administration in patients enrolled in an ongoing clinical trial (NCT04560972) (Feng et al., 2023). Preclinical work from the Netherlands Cancer Institute has identified an enzymatic pathway responsible for converting LB100 into endothall, providing a mechanistic basis for the prodrug-to-active-metabolite conversion (Dias et al., 2025).

This conversion has clinical consequences because endothall is a well-characterized environmental toxicant with a distinct and severe toxicity profile independent of its anticancer utility. In comparative *in vivo* toxicology studies of PP1 and PP2A inhibitors endothall, cantharidin, and endothall thioanhydride, endothall caused pronounced hepatotoxicity, ATP depletion, and glycogenolysis in liver tissue (Graziano and Casida, 1987; Erdodi et al., 1995). In humans, endothall is a recognized caustic agent. Regulatory toxicology assessments verified that the gastrointestinal tract is the primary target organ for endothall (Sulaeman et al., 2024).

Although free endothall is known to cause severe gastrointestinal injury, the Phase I trial of LB100 (NCT01837667) did not report such events (Table 3). The dose limiting toxicities were reversible increases in serum creatinine. Other grade 3 adverse events included anemia, dyspnea, hyponatremia, and lymphopenia. These findings suggest that controlled intravenous dosing and the prodrug strategy may reduce the direct toxicity associated with free endothall exposure (Chung et al., 2017). The prodrug design of LB100 aims to harness endothall’s strong phosphatase inhibitory activity while limiting systemic exposure to the toxicant.

**TABLE 3:** Completed and ongoing clinical studies of LB100.

Trial/Phase/status/Indication	Strategy	Key safety or pharmacokinetic findings	Efficacy
NCT01837667/Phase I; completed/Advanced solid tumors	LB100 monotherapy; intravenous administration once daily for 3 days every 21 days	Dose-limiting reversible increases in serum creatinine or reduced creatinine clearance at 3.1 mg/m^2^; recommended phase II dose: 2.33 mg/m2 daily for 3 days every 3 weeks. Other treatment-related grade 3 events included anemia, dyspnea, hyponatremia, and lymphopenia	Early/limited antitumor activity; study primarily established dose and tolerability
NCT03027388/Phase 0; completed/Recurrent gliomas/glioblastoma	Preoperative pharmacokinetic “window-of-opportunity” study	Intact LB100 in resected tumor averaged 0.31% of matched plasma concentration and was approximately 1,000-fold below the reported PP2A-inhibitory IC50	No conclusion regarding direct CNS antitumor efficacy; low tumor exposure challenges pharmacologically meaningful target engagement by intact LB100
NCT03886662/Ongoing/Low- or intermediate-1-risk myelodysplastic syndromes	LB100-containing regimen	Results not yet available	Not yet available
NCT05809830/Ongoing/Advanced soft-tissue sarcoma	LB100 plus doxorubicin	Results not yet available	Not yet available
NCT06012734/Ongoing/Metastatic microsatellite-stable colorectal cancer	LB100 plus PD-1/PD-L1-directed immunotherapy	Results not yet available	Not yet available
NCT06065462/Ongoing/Recurrent ovarian clear-cell carcinoma, peritoneal carcinoma, or fallopian-tube carcinoma	LB100 plus dostarlimab	Results not yet available	Not yet available

However, LB100 is pH-dependent and unstable, and repeated dosing has been shown to cause *in vivo* accumulation of endothall (Feng et al., 2023). Therefore, careful pharmacokinetic monitoring should be undertaken to prevent unintended toxicity in longterm or intensified regimens with LB100.

## 6 Defining the therapeutic corridor for PP2A/PP5-directed cancer therapy

The first-in-human LB100 study provides the initial clinical boundary conditions for transient PP2A/PP5-directed inhibition. LB100 was administered intravenously once daily for three consecutive days in 21-day cycles. At 3.1 mg/m^2^, dose-limiting toxicity consisted of reversible increases in serum creatinine or reductions in calculated creatinine clearance; 2.33 mg/m^2^ daily for 3 days every 3 weeks was selected as the recommended phase II dose. Grade 3 adverse events considered probably or possibly related to treatment included anemia, decreased creatinine clearance, dyspnea, hyponatremia, and lymphopenia (Chung et al., 2017).

These findings support intermittent combination development but do not by themselves define a therapeutic corridor. Here, the therapeutic corridor denotes the range of schedule, systemic and tissue exposure, target engagement, and tumor context in which short-term PP2A/PP5-directed modulation enhances anticancer treatment while maintaining acceptable systemic and neurobiological toxicity. Defining this range requires separate evaluation of intact LB100 and endothall, tumor and normal-tissue distribution, molecular target engagement, and exposure–toxicity relationships.

Current studies also reflect the combination-oriented positioning of LB100. The compound is being evaluated with doxorubicin in advanced soft tissue sarcoma (NCT05809830, Table 3), with PD-1/PD-L1 inhibitors in recurrent ovarian clear cell carcinoma and metastatic microsatellite-stable colorectal cancer (NCT06065462; NCT06012734, Table 3), and in a neuro-oncology pharmacokinetic study of LB100 in recurrent gliomas/glioblastoma tissue (NCT03027388, Table 3). A separate study in low- or intermediate-1-risk myelodysplastic syndromes extends the clinical context of LB100 beyond solid tumors (NCT03886662, Table 3). Published clinical evidence is presently limited, and ongoing studies have not yet reported mature safety, pharmacokinetic, pharmacodynamic, or efficacy results (Table 3). The CNS Phase 0 study quantified intact LB100 in resected tumor tissue; it did not establish endothall exposure in tumor, cerebrospinal fluid, or normal brain.

Thus, the clinical value of LB100 at this stage lies less in established antitumor efficacy than in its ability to define the initial human boundary conditions for PP2A/PP5-directed inhibition: a feasible dosing schedule, an early tolerability profile, a renal safety signal, and a dose range for further combination testing (Chung et al., 2017). These parameters describe the clinical tolerability of a defined regimen, but they do not fully define the therapeutic corridor. In this review, the therapeutic corridor is defined as the range of dose, schedule, and tissue exposure within which short-term PP2A/PP5-directed intervention enhances anticancer therapy while maintaining acceptable systemic and neurobiological toxicity. Such an assessment requires pharmacological attribution of the observed effects: which molecular form mediates tumor sensitization, which form contributes to toxicity, and to what extent the observed activity reflects controllable PP2A/PP5 inhibition.

The significance of this intervention will largely depend on a few key factors: (i) Achieving pharmacologically meaningful modulation of PP2A/PP5 at tolerable exposure levels (Chung et al., 2017; D’Arcy et al., 2019); (ii) Consistently enhancing tumor-cell sensitivity to chemotherapy, radiotherapy, or immunotherapy (Hong et al., 2015; D’Arcy et al., 2019); and (iii) Maintaining therapeutic window sufficiently broad to support the development of LB100 or its pharmacologically optimized analogs.

Rollema et al. showed that LB100 hydrolyzes to endothall and N-methylpiperazine, and that endothall has stronger phosphatase-inhibitory activity than intact LB100 (Rollema et al., 2025). These findings support a hydrolysis-dependent, prodrug-like interpretation of LB100 pharmacology, but they also introduce an important safety constraint. Endothall should be considered a pharmacologically active hydrolysis product that may contribute to the overall phosphatase-inhibitory exposure of LB100.

Further development of LB100, or of LB100-like analogs with related hydrolysis-dependent behavior, will require separate assessment of the parent compound, endothall, tissue exposure, target engagement, and organ-specific toxicity. This requirement does not imply that endothall is a desirable therapeutic product; rather, it defines a pharmacological liability that must be measured, controlled, or reduced in future PP2A/PP5-directed strategies.

PEBP1, known as phosphatidylethanolamine-binding protein 1/Raf kinase inhibitor protein (PEBP1/RKIP), should also be considered as a potential additional variable affecting the cellular response to LB100. PEBP1/RKIP is a cytosolic regulatory protein linked to Raf/MEK/ERK, NF-κB, and PI3K/Akt/mTOR signaling, cellular stress adaptation, and drug response. Preliminary data on LB100 suggest that PEBP1 may be associated with the pharmacological behavior of the compound, including a possible role in conversion of LB100 to endothall; this hypothesis remains preliminary and requires independent validation (Dias et al., 2025). In this context, PEBP1 should be framed as a candidate modifier of the cellular response to LB100 exposure, rather than as a proven determinant of drug binding, intracellular distribution, or compound stability. Its relevance should therefore be interpreted at the level of response modulation and pharmacological stratification, pending independent experimental validation.

The pharmacological uncertainty of LB100 is directly connected to nervous-system safety. If systemic administration produces PP2A/PP5-directed activity or endothall-related exposure outside the tumor, assessment of the therapeutic corridor should include antitumor activity, renal toxicity, and potential effects on the brain (summarized in Figure 3).

**FIGURE 3:**
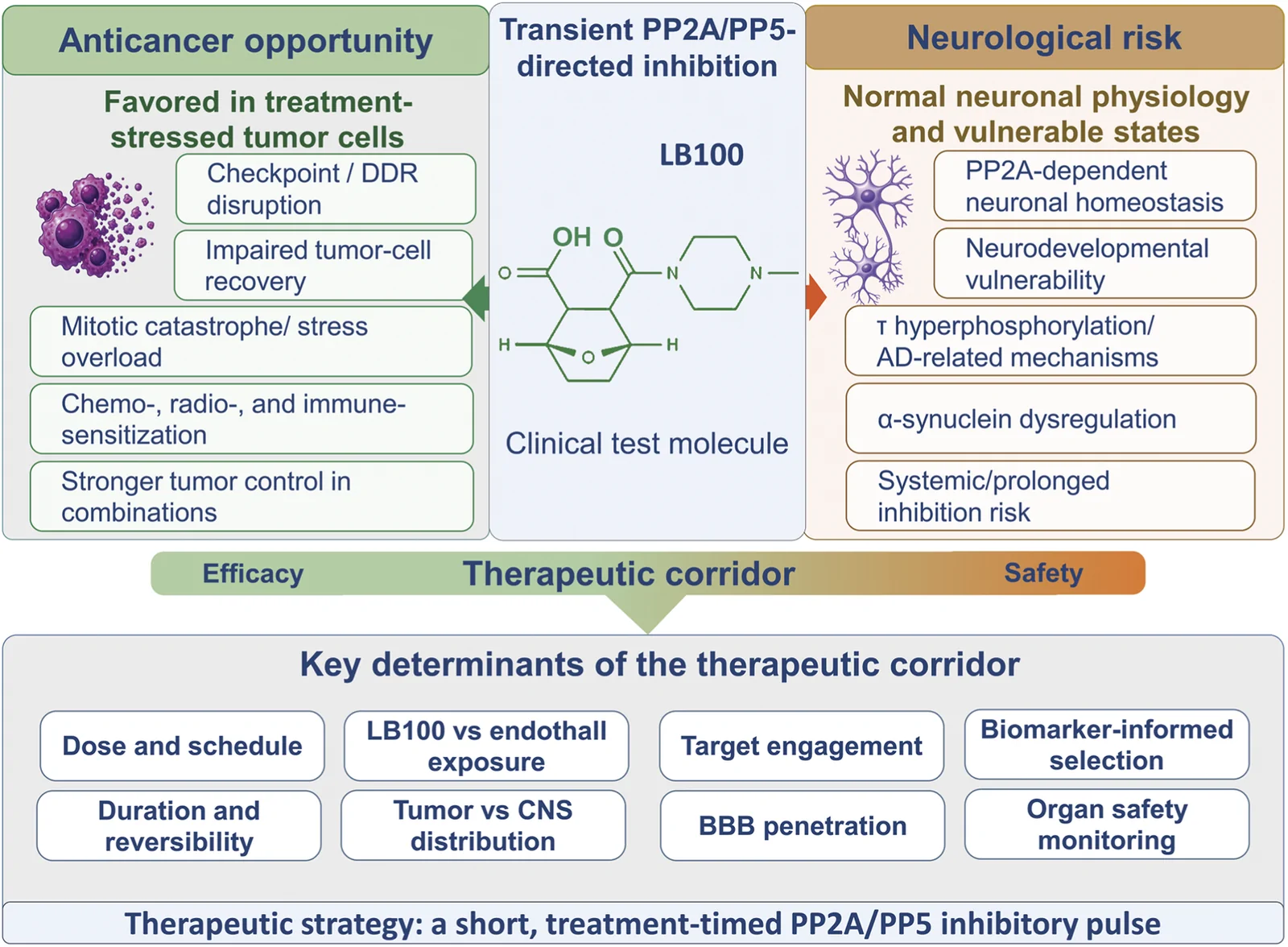
Therapeutic corridor for transient PP2A/PP5-directed inhibition: balancing anticancer potential and neurological risk. LB100 is presented as a clinical test molecule for evaluating a transient-inhibition strategy rather than as a rationale for sustained systemic PP2A suppression. The left side of the scheme illustrates the rationale for transient PP2A/PP5 inhibition in treatment-stressed tumor cells. Disruption of checkpoint control and DNA-damage adaptation may impair tumor-cell recovery, promote mitotic catastrophe and stress overload, and increase sensitivity to chemotherapy, radiotherapy, and immunotherapy. The right side of the scheme reflects the physiological dependence of the nervous system on PP2A-regulated homeostasis and potential vulnerabilities associated with neurodevelopment, tau hyperphosphorylation and Alzheimer’s disease-related mechanisms, α-synuclein dysregulation, as well as systemic or prolonged phosphatase inhibition. Key determinants of the therapeutic corridor are summarized in the lower panel of the scheme.

### 6.1 Systemic exposure and active-metabolite burden

The therapeutic corridor of LB100 cannot be defined by the administered parent-drug dose alone. LB100 undergoes pH-dependent hydrolysis in aqueous solution and *in vivo*, generating endothall and N-methylpiperazine (Rollema et al., 2025). Because endothall displays stronger phosphatase-inhibitory activity than intact LB100, pharmacologically relevant exposure should be considered as the integrated and time-dependent burden of both molecular species.

This distinction is clinically important because LB100 stability varies with pH, and circulating endothall has been detected during infusion, with evidence suggesting accumulation after repeated administration (Feng et al., 2023; Rollema et al., 2025). Endothall formation may therefore contribute to interpatient variability in target inhibition and toxicity.

Endothall is not merely an inactive degradation product: experimental studies associate it with hepatotoxicity, ATP depletion, and glycogenolysis, and regulatory assessments identify the gastrointestinal tract as a principal target organ (Graziano and Casida, 1987; Erdodi et al., 1995; Sulaeman et al., 2024). Although these toxicities were not prominent in the first-in-human LB100 study, the observed renal safety signal and the possibility of metabolite accumulation support separate quantification of LB100 and endothall. Future clinical studies should report parent and metabolite C_max_, AUC, trough concentrations, and exposure–toxicity relationships.

### 6.2 Tumor-selective target engagement and pharmacodynamic response

A clinically meaningful therapeutic corridor requires more than tolerable systemic exposure: it requires pharmacologically relevant PP2A/PP5 modulation in tumor tissue at the time when tumor cells are most dependent on checkpoint adaptation and stress-recovery pathways. This distinction is particularly important for LB100 because detectable plasma concentrations, tumor drug penetration, phosphatase target engagement, and clinical tumor sensitization are related but non-equivalent endpoints.

The proposed antitumor activity of LB100 is based on transient impairment of PP2A/PP5-dependent DNA-damage-response and checkpoint-recovery programs. In treatment-stressed tumor cells, this may be reflected by altered ATM/ATR-associated signaling, dysregulation of CHK1/CHK2 and PLK1 phosphorylation, impaired homologous-recombination repair, defective cell-cycle arrest, and increased mitotic catastrophe (Hong et al., 2015; Chang et al., 2015; Zhang et al., 2015; D’Arcy et al., 2019). The preclinical literature further indicates that the consequences may extend to modulation of WEE1/CHK1 dependency, P-glycoprotein-associated multidrug resistance, mRNA splicing, neoantigen formation, and antitumor immune responses (Gordon et al., 2015; Dias et al., 2024a; Dias et al., 2024b; Podolski-Renić et al., 2025; Ho et al., 2018). However, these effects are context dependent and should not be assumed to occur uniformly across tumor types or combination regimens.

Therefore, future clinical development should incorporate paired tumor–plasma pharmacokinetic and pharmacodynamic studies whenever feasible. Candidate tumor pharmacodynamic endpoints include markers of DNA damage, checkpoint dysregulation, aberrant mitotic entry, phosphoproteomic changes consistent with PP2A/PP5 modulation, and, in appropriate immune-combination studies, changes in tumor immunopeptidome or immune-cell composition. Demonstrating such changes in tumor tissue would be more informative than relying on administered dose or systemic safety alone, because it would directly test whether the intended mechanism is engaged within the relevant compartment.

### 6.3 Duration, timing, and reversibility of PP2A/PP5 inhibition

For LB100, the clinically relevant question is not whether PP2A/PP5 should be inhibited continuously, but whether a short, treatment-timed inhibitory pulse can maximize tumor sensitization while permitting normal-tissue recovery. The intended effect is a temporary disruption of checkpoint adaptation after a genotoxic or other therapy-induced stress, not durable systemic phosphatase suppression (Hong et al., 2015; D’Arcy et al., 2019; Chung et al., 2017).

The first-in-human study provides an initial clinical example of intermittent administration: LB100 was given intravenously once daily for three consecutive days in 21-day cycles. Dose-limiting toxicity at 3.1 mg/m^2^ included reversible increases in serum creatinine or reductions in calculated creatinine clearance, and 2.33 mg/m^2^ daily for 3 days every 3 weeks was selected as the recommended phase II dose. This schedule supports the feasibility of pulsed administration and recovery intervals; however, it does not yet establish the optimal temporal relationship between LB100 exposure, partner therapy, tumor DNA damage, and normal-tissue recovery.

The duration of functionally relevant phosphatase inhibition may not mirror the plasma half-life of intact LB100 because endothall generation can prolong phosphatase-directed activity. Consequently, schedule optimization should be based on integrated parent-drug/metabolite pharmacokinetics and time-resolved pharmacodynamic studies. Regimens should aim to maximize tumor sensitization during the window of therapy-induced stress while avoiding prolonged inhibition that could impair genomic integrity in normal proliferating tissues or increase cumulative organ toxicity. The reversibility of renal, hematological, and potential neurological effects should therefore be considered a defining property of a favorable therapeutic corridor rather than merely a secondary safety observation.

### 6.4 CNS exposure and blood–brain barrier penetration

The neurological boundary of the therapeutic corridor depends not only on the intrinsic vulnerability of neurons to PP2A disruption, but also on whether a PP2A/PP5-directed agent reaches the CNS at concentrations sufficient for meaningful target engagement. Neurodevelopmental and neurodegenerative evidence warrants caution, but it does not itself demonstrate clinically relevant inhibition of neuronal PP2A after systemic LB100 administration (Verbinnen et al., 2021; Sontag et al., 2004; Lee et al., 2011; Clark and Ohlmeyer, 2019).

In the phase 0 window-of-opportunity study in recurrent gliomas, intact LB100 concentration in resected tumor tissue averaged 0.31% of the matched plasma concentration and was approximately 1,000-fold below the reported PP2A-inhibitory IC50 (Burton et al., 2025). Under the conditions studied, these findings suggest limited direct target engagement by intact LB100 in brain-tumor tissue.

This observation is reassuring but should not be interpreted as proof of complete neurological safety. Brain-tumor tissue is not identical to normal brain parenchyma; BBB integrity may differ among tumor types, anatomical regions, and patients; and the CNS distribution and pharmacological activity of endothall have not been equivalently established. Moreover, the potential impact of repeated treatment, altered barrier function after radiotherapy or surgery, and concomitant therapies requires further assessment. Therefore, future studies should distinguish plasma exposure, tumor exposure, normal-brain or cerebrospinal fluid (CSF) exposure where feasible, and CNS pharmacodynamic effects. Neurological examination and longitudinal assessment of cognitive symptoms should be proportionate to the anticipated CNS exposure and the duration of treatment.

In the first-in-human Phase I study of LB100 in patients with advanced solid tumors, the reported dose-limiting toxicity was reversible renal dysfunction, and the treatment-related grade 3 adverse events were anemia, decreased creatinine clearance, dyspnea, hyponatremia, and lymphopenia; neurological adverse events were not reported among these key safety findings (Chung et al., 2017). This absence is encouraging but should be interpreted cautiously. The study was designed primarily to define dose-limiting toxicity and tolerability in a small, heterogeneous population, rather than to detect infrequent, delayed, or subtle neurological effects or to assess cognition longitudinally. Moreover, the duration of exposure was limited and CNS exposure to endothall was not characterized. Consequently, the available Phase I data do not indicate an obvious neurological safety signal, but they are insufficient to exclude neurological risk during prolonged treatment, intensified schedules, or clinical settings associated with altered BBB integrity.

### 6.5 Future directions in patient selection and translational monitoring

The clinical utility of PP2A/PP5-directed inhibition is likely to depend on selecting tumors in which transient disruption of stress-adaptation pathways produces a disproportionate therapeutic effect. Rational candidates may include tumors with high replication stress, chromosomal instability, impaired homologous-recombination repair, dependence on WEE1/CHK1 or PLK1 signaling, substantial treatment-induced DNA damage, or evidence of PP2A-pathway deregulation (Chang et al., 2015; Zhang et al., 2015; Hong et al., 2015; D’Arcy et al., 2019; Khanna et al., 2013). These hypotheses require prospective validation but provide a more coherent development framework than unselected administration of a broadly acting phosphatase inhibitor.

In glioblastoma models, LB100-mediated sensitization to temozolomide was reported in a p53-mutant setting, consistent with the possibility that PP2A inhibition can perturb checkpoint control when canonical p53-dependent responses are compromised (Hong et al., 2015). However, the available evidence does not establish *TP53* status as a stand-alone predictor of response to PP2A/PP5 inhibition. Similarly, RAS/MAPK-driven tumors are mechanistically relevant because PP2A holoenzymes regulate the amplitude and duration of MAPK signaling; however, the direction of the effect depends on holoenzyme composition and should not be inferred from RAS mutation status alone (Adams et al., 2005; Lao et al., 2007; Sangodkar et al., 2017; Johnson et al., 2024).

Alterations in the PP2A pathway may provide an additional level of biological stratification. Cancer-associated *PPP2R1A* mutations impair B-subunit binding and disrupt PP2A holoenzyme assembly, thereby altering downstream signaling outputs; other recurrent mechanisms include abnormalities in *PPP2R1B*, *PPP2R2A*, *PPP2R4/PTPA*, SET, CIP2A, PME-1, and PP2A catalytic-subunit modification (Calin et al., 2000; Shih et al., 2011; Baldacchino et al., 2014; Beca et al., 2015; Sents et al., 2017; Ribaudo et al., 2025; Pavic et al., 2023; Johnson et al., 2024). These alterations may influence residual PP2A dependency, signaling architecture, and the cellular response to pharmacological PP2A/PP5 inhibition. However, their predictive value for LB100 has not yet been prospectively validated and they should presently be considered mechanistically motivated biomarkers for evaluation in translational studies.

PEBP1/RKIP may represent one potential response-modifying variable. Preliminary evidence links PEBP1 to the pharmacological behavior of LB100 and possibly to its conversion to endothall, but this relationship remains unconfirmed. It should therefore be evaluated as an exploratory biomarker rather than presented as an established determinant of LB100 sensitivity, intracellular distribution, or metabolism (Dias et al., 2025).

A translational monitoring strategy should integrate four domains: (i) pharmacokinetics of both LB100 and endothall in plasma and, where possible, tumor tissue; (ii) tumor pharmacodynamic evidence of checkpoint disruption, DNA-damage persistence, aberrant mitotic entry, or immune remodeling; (iii) biomarker assessment of tumor stress and PP2A/PP5-pathway dependence; and (iv) organ-specific safety monitoring, including renal function, hematologic parameters, electrolytes, hepatic markers, gastrointestinal toxicity, and neurologic assessment when CNS exposure is plausible. Such integrated monitoring would allow the therapeutic corridor to be refined empirically and would distinguish regimens that produce tumor-selective sensitization from those that merely increase systemic phosphatase-inhibitory burden.

In this framework, the future of LB100 and related compounds depends less on establishing a universally tolerated dose than on identifying a pharmacologically measured, schedule-specific, and biologically selected window in which tumor benefit exceeds systemic and neurobiological risk.

## 7 Conclusion

PP2A-targeted therapy illustrates that the role of PP2A in tumor initiation may differ from its therapeutic role in established, treatment-stressed tumors. Although PP2A generally restrains oncogenic signaling, transient PP2A/PP5-directed inhibition with LB100 may sensitize selected tumors to chemotherapy, radiotherapy, or immunotherapy by interfering with checkpoint adaptation and stress recovery.

The feasibility of this strategy depends on defining a measurable therapeutic corridor that integrates parent-drug and endothall exposure, schedule, tumor and normal-tissue distribution, target engagement, tumor-specific stress dependence, and reversibility of systemic toxicity. Low intact LB100 penetration in resected recurrent-glioma tissue is encouraging with respect to direct CNS target engagement under the tested conditions, but it does not exclude neurological risk in settings of altered BBB integrity, repeated treatment, or metabolite exposure.

Future studies should prioritize paired tumor–plasma pharmacokinetics, separate measurement of LB100 and endothall, direct pharmacodynamic evidence of PP2A/PP5 modulation, schedule optimization, and biomarker-guided patient selection. The value of PP2A/PP5 inhibition is therefore unlikely to lie in sustained systemic phosphatase suppression, but rather in short, quantifiable, and tumor-informed perturbation of stress-adaptation pathways within combination regimens.
